# Assessing validity and reliability of the Benefit Finding Scale in Italian people with multiple sclerosis and their caregivers

**DOI:** 10.1007/s10072-025-08776-6

**Published:** 2026-01-27

**Authors:** Rosalba Rosato, Andrea Giordano, Beatrice Biolzi, Clara Grazia Chisari, Monica Falautano, Monica Grobberio, Claudia Niccolai, Erika Pietrolongo, Maria Esmeralda Quartuccio, Rosa Gemma Viterbo, Antonella Delle Fave, Marta Bassi

**Affiliations:** 1 Department of Psychology, University of Turin, Turin, Italy; 2 Neurology, Public Health, Disability Unit, Fondazione IRCCS Istituto Neurologico C. Besta, Milan, Italy; 3 Multiple Sclerosis Center, Neurology Unit, Hospital of Vaio, Fidenza, Italy; 4 Dipartimento di Scienze Mediche e Chirurgiche e Tecnologie Avanzate, GF Ingrassia, University of Catania, Catania, Italy; 5 Psychological Service - Neurological and Neurological Rehabilitation Units, IRCCS San Raffaele, Milano, Italy; 6 Laboratory of Clinical Neuropsychology, Psychology Unit, ASST Lariana, Como, Italy; 7 IRCCS Don Gnocchi Foundation, Florence, Italy; 8 Department of Neurosciences, Imaging and Clinical Sciences, University G. d’Annunzio, Chieti, Italy; 9 Department of Neuroscience, San Camillo-Forlanini Hospital, Rome, Italy; 10 Department of Basic Medical Sciences, Neurosciences and Sense Organs, University of Bari, Bari, Italy; 11 Department of Pathophysiology and Transplantation, Università di Milano, Milan, Italy; 12 Department of Biomedical and Clinical Sciences, Università di Milano, Milan, Italy

## Abstract

**Background:**

The Benefit Finding Scale (BFS) is a 17-item measure assessing the perception of positive contributions to one’s life deriving from stressful and life-threatening conditions such as illnesses. We aimed to investigate construct validity (structural validity, measurement invariance between sub-samples, and convergent/divergent validity) and reliability (internal consistency) of the Italian version of the BFS in persons with multiple sclerosis (PwMS) and their caregivers.

**Methods:**

We used confirmatory factor analysis (CFA) to assess structural validity in terms of the proposed three-factor structure of the BFS. We performed multi-group CFA to assess measurement invariance between PwMS and their caregivers. To assess convergent/divergent validity, we calculated correlations of the BFS subscales with instruments measuring affect (PANAS), life satisfaction (SWLS), social support (MSPSS), depression (BDI-II), and quality of life (SF-36 and MSQoL-54). To appraise internal consistency, we calculated Cronbach’s alpha.

**Results:**

A total of 1359 PwMS and their caregivers completed the study. The three-factor structure of the BFS showed good fit (RMSEA 0.06; CFI 0.92; SRMR 0.05). Configural, metric and scalar invariance were confirmed. Convergent/divergent validity was supported. The BFS showed good internal consistency for ‘Acceptance and adjustment’ (alpha 0.82), ‘Family relations and sense of connectedness’ (alpha 0.84) and ‘Personal growth and authenticity’ (alpha 0.85).

**Conclusions:**

Results support the BFS as a valid and invariant three-factor measure of benefit finding among Italian PwMS and their caregivers. This scale use in clinical practice could help health professionals track participants’ experience of positive changes under adverse circumstances, as assets in managing stress and promoting illness adjustment.

## Introduction

Multiple sclerosis (MS) is a chronic, disabling neurological disease affecting 2.3 million people worldwide, predominantly young adults [1]. Approximately 85% of persons with MS (PwMS) are initially diagnosed with the relapsing-remitting form [2], and around half will eventually develop secondary progressive MS, with transition commonly reported within 10–20 years from disease onset in natural history studies [3, 4]. The limited effectiveness of current disease-modifying therapies, their unknown long-term effects, the prognostic uncertainty, and the wide symptom variety (e.g., fatigue, depression, physical and cognitive impairments) have a negative effect on quality of life (QoL) yet remaining unrecognized in clinical practice [5]. These issues affect PwMS as well as their caregivers, who can feel distress, anxiety and depressive symptoms [6, 7]. At the same time, it is well-recognized that PwMS and caregivers can also identify positive changes related to living with MS or caring for a PwMS. Particularly, such changes are related to well-being dimensions, such as a sense of personal growth, deeper spirituality and straightened family relationships [8, 9].

Different theoretical frameworks have been proposed to explain the occurrence of this phenomenon, such as benefit finding (BF) [10], meaning making [11], post-traumatic growth [12], and hedonic adaptation to positive and negative experiences [13].

In our study, we referred to BF as a coping strategy, which was defined as “finding something good resulting from the experience or coping process of a stressful event” [14] or condition, such as having MS or providing care to a PwMS. Particularly, BF is conceived as a meaning-based strategy that can restore meaningfulness, purpose in life and a redefined self in the face of adversities [9, 15].

Among PwMS and their caregivers, BF was shown to play an important role in the stress adaptation process, promoting creativity, problem-solving, protection against negative emotions, such as anxiety and depression, as well as enhanced positive affect, and life satisfaction [9, 15–18]. It was further shown to increase dyadic adjustment and to promote readiness to provide care among caregivers [15].

Clinical interest in BF has increased in recent years, leading to the development of multiple assessment instruments. In the MS field, disease-specific instruments were developed separately for PwMS and caregivers [9, 15]. However, they are long (67 and 55 items, respectively) and show limited psychometric robustness, which may hinder their feasibility and utility in both research and clinical practice.

In oncology, findings from a systematic review showed that 17 different BF instruments have been identified. Nevertheless, further research is needed to improve their psychometric properties [19]. Among these 17 instruments, the Benefit Finding Scale (BFS) has demonstrated good internal consistency, but evidence on its construct validity is mixed, with either unidimensional [20–23] or multidimensional [24–26] structures reported. Particularly, Li et al. (2017) identified three factors tapping into ‘Acceptance’, ‘Improved relationship’, and ‘Personal growth’ [26].

As far as we know, no validation studies were conducted to evaluate the psychometric properties of the BFS in Italy among PwMS and their caregivers. A version of the BFS that is demonstrably valid and reliable for both groups would enable comparative assessments and streamline research across different health conditions in the Italian setting. Therefore, our aim was to assess the construct validity (structural validity, measurement invariance between subgroups, convergent/divergent validity) and reliability (internal consistency) of the BFS in a large sample of Italian PwMS and their caregivers.

## Methods

### Study design

This cross-sectional study included PwMS involved in a larger research project along with their caregivers and healthcare professionals from eight MS centres in Italy [27]. The protocol was approved by the local ethical committees, and the study was carried out in accordance with the Good Clinical Practice and Declaration of Helsinki principles.

For PwMS, inclusion criteria were age 18–55 (representing the productive phase in people’s lives), a clinically definite MS diagnosis (McDonald’s revised criteria) received at least 3 years earlier, having a caregiver. Exclusion criteria were presence of psychiatric and/or other neurological disorders, experiencing the active phase of disease, Extended Disability Status Scale (EDSS) score > 8 [28], and severe cognitive impairment in logical abilities and comprehension, with at least one score below the cutoff point (16th percentile; equivalent score 0–1) at the Raven Coloured Progressive Matrices or at the Token Test. No specific criteria were applied to caregivers’ recruitment [27].

All participants were administered a battery of self-reported psychological questionnaires assessing various well-being and ill-being dimensions, as well as personal and social resources in facing MS. Details about the data collection procedures are reported elsewhere [27]. All participants gave written informed consent before study enrolment.

## Instruments

### Benefit Finding Scale

The Benefit Finding Scale (BFS) is a 17-item measure evaluating the perception of positive contributions to one’s life deriving from stressful and life-threatening experiences [29, 30]. Responses are provided through a 1 (I disagree a lot) to 5 (I agree a lot) scale; higher scores reflect higher perceived benefit. The items begin with “Having MS has…” for PwMS, and “Providing care for PwMS has…” for caregivers. For each of the three subscales (Acceptance, Improved relationship, and Personal Growth) [27], scores are obtained by summing up the relevant items. This scale has been validated in both people with cancer and caregivers [20–25].

## Cross-cultural adaptation

Following the International Society for Pharmacoeconomics and Outcomes Research Translation-Cultural Adaptation Task Force Guidelines [31, 32], we cross-culturally adapted the BFS in four subsequent steps:

1. Forward translation: one qualified translator, expert in psychology, and living in Italy, produced her independent translation. A panel consisting of the translator, a senior researcher in psychology and a young research assistant reviewed the forward translation (meeting 1) and a reconciled version was produced. Besides the professional translator, all the panel members were fluent in English.
2. Backward translation: the reconciled version generated in step 1 was translated back into US English by the qualified translator. The backward translation was produced without access to the original version.
3. Pre-final version: the same panel members participating in step 1 met again (meeting 2) to compare the backward translation with the original and add further refinements to the Italian version. Differences were resolved by consensus, and a pre-final version was agreed upon.
4. Questionnaire refinement: The questionnaire was refined and proof-read.

## Positive affect and negative affect schedule

The Positive Affect and Negative Affect Schedule (PANAS) includes 10 items assessing positive affect (PA; e.g., interested) and 10 items measuring negative affect (NA; e.g., distressed) [33]. Respondents are asked to indicate to what extent they felt each item “during the last two weeks” on scales from 1 (very slightly or not at all) to 5 (extremely). For PA and NA, mean scores are calculated by averaging their respective items. Higher scores indicate more intense affect states.

### Satisfaction with life scale

The Satisfaction with Life Scale (SWLS) [34] evaluates participants’ agreement from 1 (strongly disagree) to 7 (strongly agree) on five statements about their overall life satisfaction. A total score is obtained by summing up all the item scores, with higher scores indicating higher satisfaction with life levels.

## Beck depression Inventory-II

The Beck Depression Inventory-II (BDI-II) [35] comprises 21 items rating perceived depressive symptoms on 0–3 scales. A total score is obtained by averaging all items, with higher values indicating higher symptom levels.

## SF-36

The SF-36 is a widely used self-reported generic health-related quality of life (HRQoL) measure consisting of 36 items tapping into eight domains: physical function, physical role limitations, emotional role limitations, social function, pain, vitality, general health, and mental health. Two composite scores (Physical and Mental Health) can be calculated, with higher scores indicating higher HRQoL levels [36]. In the present study, this measure was completed by the caregivers.

### MSQoL-54

The MSQoL-54 measures HRQoL of PwMS. It comprises the generic SF-36 items, plus 18 MS-specific items [37]. As for SF-36, two composite scores (Physical and Mental Health) are derived by combining scores of the relevant subscales. Higher scores indicate higher HRQoL levels.

For comparative reasons with caregivers’ scores, the calculation of the Physical and Mental Health composite scores for patients were derived exclusively from the SF-36 items.

### Multidimensional scale of perceived social support (MSPSS)

The MSPSS measures perceived social support offered by family, friends and significant others with 12 items on scales ranging from 1 (strongly disagree) to 5 (strongly agree) [38]. PwMS were asked to what extent they received support in managing their health condition, while caregivers were asked to what extent they received support in their caring role to PwMS. A total score is obtained by averaging all items, with higher values indicating higher social support levels.

### Statistical analysis

Variables were summarized using both counts and percentages, means and standard deviations (SD), or medians and minimum–maximum ranges.

#### Factor structure and measurement invariance

As a preliminary analysis, we applied confirmatory factor analysis (CFA) with robust maximum likelihood estimator [39] to test the three-factor structure of the BFS proposed by Li et al. (2017). The model included three latent dimensions: ‘Acceptance’ (3 items), ‘Improved relationship’ (5 items), and ‘Personal growth’ (9 items) [27]. The resulting model was then used to test multi-group measurement invariance between PwMS and their caregivers.

Three increasingly restricted levels of measurement invariance were assessed. First, configural invariance was tested to verify whether the same factorial structure—namely the number of latent factors and the pattern of item–factor loadings—was shared across groups. Second, metric invariance was assessed by constraining factor loadings to be equal across groups, to examine whether items contributed to the latent constructs to the same extent in PwMS and caregivers. Finally, scalar invariance was tested by additionally constraining item intercepts to equality across groups, to evaluate whether observed group differences in item scores could be attributed to differences in the underlying latent constructs -conditions necessary for meaningful comparisons of latent means.

In evaluating the factor solution and configural invariance, model fit was evaluated according to established criteria. Specifically, model fit was considered acceptable when the χ²/df ratio was lower than 3, the root mean square error of approximation (RMSEA) was below 0.08, the comparative fit index (CFI) was equal to or greater than 0.90, and the standardized root mean square residual (SRMR) was equal to or lower than 0.08 [40, 41]. Akaike Information Criterion (AIC) and Bayesian Information Criterion (BIC) values were used to compare competing models, with lower values indicating better fit. Metric invariance was evaluated by examining changes in model fit indices relative to the configural model: a decrease in CFI greater than 0.01, accompanied by a change in RMSEA of at least 0.015 or a change in SRMR of at least 0.03, was considered indicative of lack of metric invariance. When assessing scalar invariance, the same cutoff values for changes in CFI and RMSEA were applied, whereas a more stringent cutoff of 0.01 was adopted for changes in SRMR [42, 43].

### Reliability and validity

Internal consistency was assessed using Cronbach’s alpha (benchmark value > 0.70) [44].

Pearson’s correlation coefficients were used to investigate convergent/divergent validity. According to the literature [10], we hypothesized the BFS to have small positive correlation with positive affect (PA), satisfaction with life (SWLS), social support (MSPSS), and the Mental Health Composite (MHC) of HRQoL, no correlation/small negative correlation with negative affect (NA), depressive symptoms (BDI-II), and the Physical Health Composite (PHC) of HRQoL. All the analyses were performed with SAS version 9.4 and MPLUS version 8. Significance level was set at 0.05.

## Results

### Participants

A group of 680 PwMS and 679 caregivers participated in this study. PwMS were mainly women (64.4%), with a mean age of 40.1 years (SD 9.9). The majority had relapsing-remitting MS (69.4%), and their mean disability level was mild‐to‐moderate (mean EDSS 3.3, SD 2.1). Half of caregivers were women (51.2%), with a mean age of 46.5 years (SD 12.8). Caregivers were primarily spouses (61.6%), 22.5% were parents (mainly mothers), 6.5% siblings, 5.4% children, 2.5% friends and 1.5% other relatives. On average, they had taken care of PwMS for 8.6 years (SD 5.8). Participants’ characteristics are fully reported in Table 1.

**Table 1:** Characteristics of people with MS and their caregivers

	PwMS (*N* = 680)	Caregivers (*N* = 679)
Age^a^	40.1 (9.9)	46.4 (12.8)
Gender (% women)	64.4	51.2
Education (%)		
Elementary school	0.7	4.0
Middle school	20.7	26.0
High school	52.9	46.8
University	25.6	23.2
Employment (%)	60.1	68.2
Civil status (%)		
Married/cohabiting/engaged	76.5	87.1
Single/divorced/widowed	23.5	12.9
MS type (%)		
Relapsing-remitting	69.4	-
Primary progressive	5.7	-
Secondary progressive	24.9	-
Disease duration^a^ (years)	11.5 (6.9)	-
Disability level^a^ (EDSS)	3.3 (2.1)	-
% mild (0 to 3.0)	54.1	-
% moderate (3.5 to 6.0)	32.5	-
% severe (> 6.5)	13.4	-
Relation with PwMS (%)	-	
Spouses	-	61.6
Parents	-	22.5
Siblings	-	6.5
Children	-	5.4
Friend	-	2.5
Other relative (e.g., cousin)	-	1.5
Caregiving time (years)^a^	-	8.6 (5.8)

*EDSS Expanded Disability Status Scale; MS* multiple sclerosis; *PwMS* people with multiple sclerosis

^a^Means (SD) are reported for these variables

Distribution of the self-reported outcome measures in addition to the BFS, and comparisons between PwMS and caregivers are described in Table 2.

**Table 2:** Distribution of the convergent/divergent validity measure scores, and comparisons between people with MS and caregivers

	Total (*N* = 1359)		PwMS (*N* = 680)		Caregivers (*N* = 679)	
	Mean (SD)	Median (Min-Max)	Mean (SD)	Median (Min-Max)	Mean (SD)	Median (Min-Max)
PA	3.0 (0.7)	3.1 (1.0–5.0)	2.9 (0.8)	3.0 (1.0-4.9)	3.1 (0.7)	3.2 (1.1-5.0)
NA	2.0 (0.7)	1.9 (1.0–5.0)	2.1 (0.8)	2.0 (1.0–5.0)	1.9 (0.7)	1.8 (1.0-4-5)
PHC^1,2^	53.7 (9.0)	55.2 (23.6–75.5)	55.3 (9.4–56.2)	65.2 (26.7–75.5)	52.2 (8.2)	54.7 (23.7–67.5)
MHC^1^	54.5 (16.5)	51.8 (13.3–89.9)	63.3 (17.2)	66.6 (15.4–89.9)	45.6 (9.5)	47.1 (13.3–65.2)
SWLS	21.5 (7.2)	22.0 (5.0-35-0)	20.1 (7.3)	20.0 (5.0–35.0)	22.9 (7.3)	20.0 (5.0–35.0)
BDI-II	8.4 (8.1)	6.0 (0–57.0)	10.4 (8.7)	8.5 (0–51.0)	6.4 (6.9)	5.0 (0–57.0)
MSPSS	3.7 (0.9)	3.8 (1.0–5.0)	4.0 (0.7)	4.2 (1.0–5.0)	3.4 (0.9)	3.6 (1.0–5.0)

*BDI-II* Beck depression inventory-II; *MHC* mental health composite; *MSPSS* multidimensional scale of perceived social support; *NA* negative affect; *PA* positive affect; *PwMS* people with multiple sclerosis; *SWLS* satisfaction with life scale; *PHC* physical health composite; *SD* standard deviation

^1^The calculation of the Physical and Mental health composite scores was derived exclusively from the SF-36

^2^Missing replies for PHC (PwMS): *N* = 4

### Factor structure

**Fig. 1:**
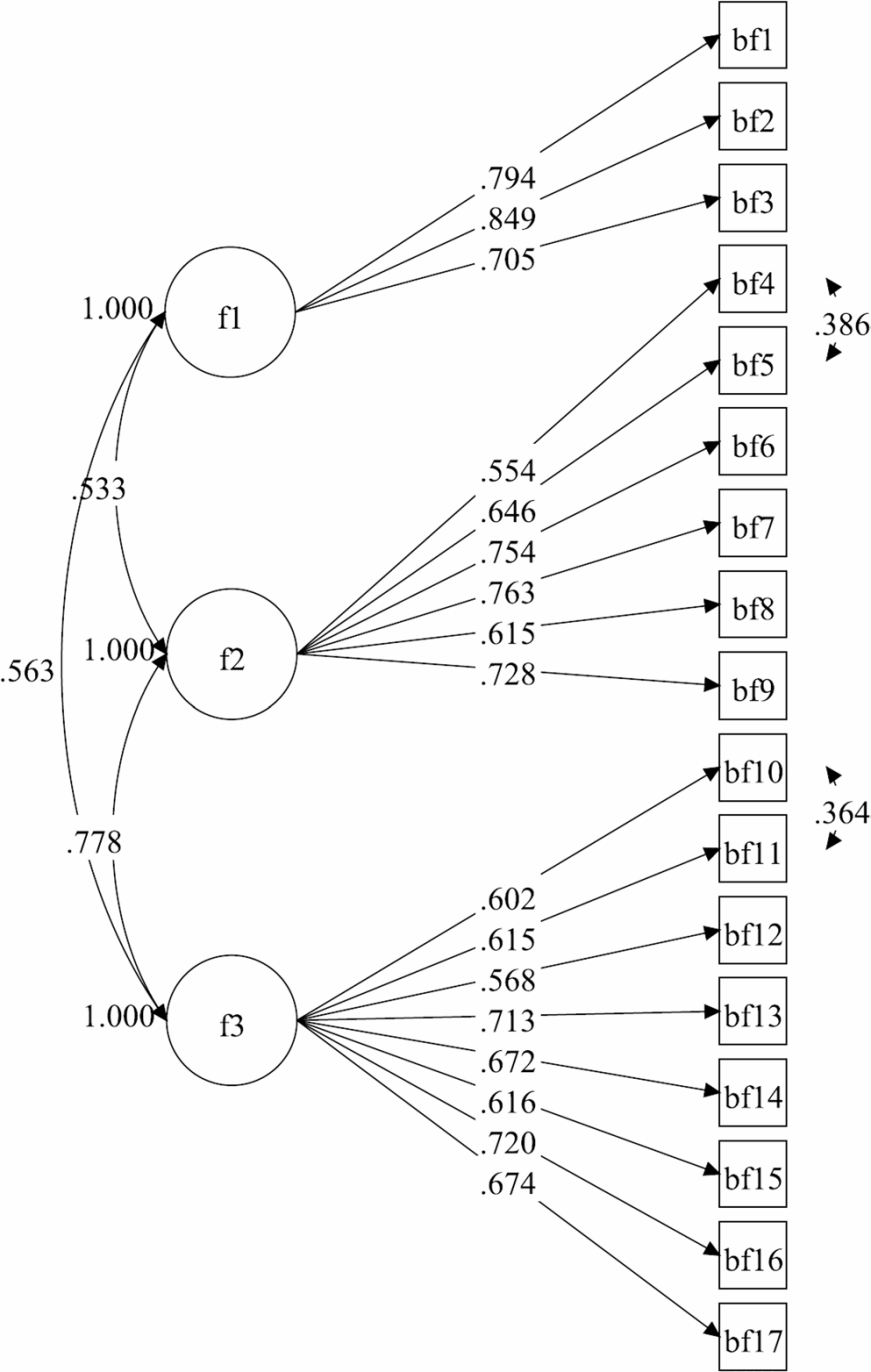
Loadings of 17 items for the total sample (*N* = 1359). Factor structure of the Benefit Finding Scale. All estimates are statistically significant *p* < 0.01. F1, Acceptance and adjustment; F2, Family relations and sense of connectedness; F3, Personal growth and authenticity

Before testing the three-factor structure of the BFS proposed by Li et al., two residual correlations between item 4 (*‘Brought my family closer together’*) and 5 (*‘Make me more sensitive to family issues’*), and between item 10 (*‘Taught me to be patient’*) and 11 (*‘Lead me to deal better with stress and problems’*) were added (Fig. 1). Then, the CFA was conducted and results showed good fit (RMSEA 0.06; CFI 0.92; SRMR 0.05). The first factor was fully consistent with that of Li et al. (2017) and included three items (items 1–3). To reflect semantic content, we slightly revised the factor name from Acceptance to ‘Acceptance and adjustment’. Concerning the second and third factors, findings were almost completely consistent with those of Li et al., but for item 9 (*‘Made me more aware and concerned for the future of all human beings’*) that was included in the second factor (originally labelled as Improved relationships) instead of the third factor (Personal growth). Consequently, factor 2 comprised six items (items 4–9) and was named ‘Family relations and sense of connectedness’. Factor 3 included eight items (items 10–17) and was named ‘Personal growth and authenticity’.

Factor loadings for the BFS are reported in Fig. 1. All estimates are large and statistically significant.

### Measurement invariance

First, we assessed the three-factor model separately in the PwMS and caregiver subsamples. Fit indices were good for both PwMS (RMSEA 0.063; CFI 0.913; SRMR 0.054) and their caregivers (RMSEA 0.054; CFI 0.936; SRMR 0.045). Then, we assessed configural invariance on the total sample: The fit indices were satisfactory (RMSEA 0,059; CFI 0.925; SRMR 0.050), indicating that there were the same number of factors and the same patterns of factor loadings across PwMS and their caregivers (Table 3). We then assessed metric invariance. Our findings show that the fit indices were satisfactory (ΔRMSEA − 0.001; ΔCFI − 0.001; ΔSRMR 0.004), suggesting the invariance of factor loadings across PwMS and their caregivers. Further, we investigated scalar invariance. Again, the model fitted the data well (ΔRMSEA 0.001; ΔCFI − 0.009; ΔSRMR 0.004), showing that both loadings and intercepts were equal across PwMS and their caregivers.

**Table 3:** Benefit Finding Scale measurement invariance across people with MS and caregivers

Models		Number free parameters	AIC	BIC	*Χ*^2 °^/df	Goodness-of-fit			Comparison		
						RMSEA	CFI	SRMR	ΔRMSEA	ΔCFI	ΔSRMR
	Subgroups										
M1	People with MS (N=680)	56	31365.62	31618.85	425.97/114	0.063	0.913	0.054			
M2	Caregivers (N=679)	56	28932.05	29185.21	343.82/114	0.054	0.936	0.045			
	Multigroup invariance										
M3	Configural invariance	112	60297.67	60881.69	768.18/228	0.059	0.925	0.050			
M4	Metric invariance	98	60289.30	60800.32	789.13/242	0.058	0.924	0.054	-0.001	-0.001	0.004
M5	Scalar invariance	84	60352.10	60790.12	869.12/256	0.059	0.915	0.058	0.001	-0.009	0.004

*AIC* Akaike information criterion; *BIC* Bayesian information criterion; *CFI* comparative ft index; *DF* error degree of freedom; *MS* multiple sclerosis; *RMSEA* root mean square error of approximation; *SRMR* standardized root mean square residual

°*p* value < 0.001

All models account for error correlation between items 4/5 and items 10/11

Descriptive values of the BFS subscales for the whole sample and each subsample are reported in Table 4.

**Table 4:** Means and standard deviations of the benefit finding subscale scores for the total sample

Subscale	Total (*N* = 1359)		PwMS (*N* = 680)		Caregivers (*N* = 679)	
	*Mean (SD)*	*Median (Min-Max)*	*Mean (SD)*	*Median (Min-Max)*	*Mean (SD)*	*Median (Min-Max)*
Acceptance and adjustment	3.7 (0.9)	4.0 (1.0–5.0)	3.7 (0.9)	4.0 (1.0–5.0)	3.8 (0.8)	4.0 (1.0–5.0)
Family relations and sense of connectedness	3.4 (0.8)	3.5 (1.0–5.0)	3.3 (0.9)	3.3 (1.0–5.0)	3.5 (0.8)	3.7 (1.0–5.0)
Personal growth and authenticity	3.4 (0.8)	3.5 (1.0–5.0)	3.4 (0.8)	3.5 (1.0–5.0)	3.4 (0.8)	3.5 (1.0–5.0)

*MS* multiple sclerosis; *PwMS* people with multiple sclerosis; *SD* standard deviation

### Internal consistency

The BFS showed good internal consistency for ‘Acceptance and adjustment’ (alpha 0.82), ‘Family relations and sense of connectedness’ (alpha 0.84) and ‘Personal growth and authenticity’ (alpha 0.85).

### Convergent/divergent validity

As hypothesized, all the BFS subscale scores had small positive correlations with positive affect (ranges 0.15–0.19), satisfaction with life (ranges 0.15–0.19), and social support (ranges 0.15–0.28).

Consistently with our hypotheses, we found no/small negative correlations between negative affect and BFS Acceptance and adjustment subscale score only (-0.06). Further, we found no/small negative correlations between depressive symptoms and two BFS subscale scores (i.e., Acceptance and adjustment, and Family relations and sense of connectedness) (-0.09 and − 0.06 respectively). By contrast, we found no/negative correlation between the Mental and Physical Health Composite and BFS (Table 5).

**Table 5:** Pearson correlation (ρ) between benefit finding subscale scores and the convergent/divergent validity measures for the total sample (*N* = 1359)

	Acceptance and adjustment		Family relations and sense of connectedness		Personal growth and authenticity	
	ρ	95% CI	ρ	95% CI	ρ	95% CI
PA	0.15	0.10, 0.20	0.14	0.09, 0.20	0.19	0.14, 0.24
NA	-0.06	-0.11, -0.008	0.004*	-0.05, 0.06	-0.03*	-0.08, 0.02
PHC^1^	0.03*	-0.02, 0.08	-0.07	-0.12, -0.02	-0.05*	-0.10, 0.003
MHC^1^	0.02*	-0.03, 0.08	-0.07	-0.12, -0.01	0.05*	-0.003, 0.10
MSPSS	0.15	0.09, 0.19	0.19	0.13, 0.23	0.28	0.23, 0.33
BDI-II	-0.09	-0.15, -0.04	-0.06	-0.11, -0.007	-0.08*	-0.13, 0.02
SWLS	0.15	0.09, 0.20	0.19	0.14, 0.24	0.19	0.14, 0.24

*95% CI* 95% Confidence Interval; *BDI-II* Beck Depression Inventory-II; *MHC* mental health composite; *MSPSS* multidimensional scale of perceived social support; *NA* negative affect; *PA* positive affect; *PHC* physical health composite; *SD* standard deviation; *SWLS* satisfaction with life

^1^The calculation of the Physical and Mental Health composite scores was derived exclusively from the SF-36

All *p* values are < 0.05 except for*=ns

## Discussion

We investigated the validity and reliability of the BFS in 1359 PwMS and their caregivers. Our findings showed that the three-factor structure proposed by Li et al. (2017) was confirmed. We slightly revised the factor naming to better reflect item content. These changes were conceptually motivated and aimed to improve the interpretative clarity and theoretical consistency of the subscales. Specifically, ‘Acceptance’ was reframed as ‘Acceptance and adjustment’ to encompass both cognitive acknowledgment of MS and active adaptive processes; ‘Family relations’ became ‘Family relations and sense of connectedness’ to capture broader relational and social dimensions beyond the immediate family context; and ‘Personal growth’ was revised as ‘Personal growth and authenticity’ to reflect both developmental changes and a deeper alignment with personal values.

Regarding the other psychometric properties, the BFS showed good internal consistency across all the three subscales (all values > 0.70 threshold), and measurement invariance between PwMS and caregivers, supporting its applicability across the two populations. Also, convergent/divergent validity was supported.

Our findings are consistent with those in oncology [20, 21, 27], by confirming the excellent internal consistency and the fact that the BFS is a multidimensional construct, as confirmed by the identification of the three-factor structure. Furthermore, measurement invariance was supported suggesting that the BFS has the same meaning and the same parameters in PwMS and their caregivers, thus allowing cross-group comparisons.

Convergent/divergent validity was supported by the confirmation of our hypotheses: all the BFS subscale scores had small positive correlations with positive affect, satisfaction with life, and social support. Additionally, we found no/small negative correlations between relevant BFS subscales and negative affect and depressive symptoms. These results are consistent with the Folkman’s coping model in which the BF is conceived as a coping strategy which can further promote psychological resilience and enhance resource mobilisation [16].

This study has important implications for clinical practice and research. First, it could be crucial to provide clinicians with valuable insights into PwMS and caregivers’ psychosocial resources, by identifying key areas in which positive changes can occur during the disease course or caregiving. Further, the BFS can be used as an outcome measure to assess the efficacy of interventions aiming to promote positive adjustment to MS in individual, group or family-based programs, as well as multidisciplinary rehabilitation [9, 15, 17, 18].

The relevance of the BFS across conditions could allow for comparisons between MS and other diseases, contributing to better understanding the BF in long-term illness adjustment [10].

A few limitations should be reported. First, we used a cross-sectional design, thus precluding conclusions about causal relationships or the stability of BF over time. To overcome this limitation, future studies should use longitudinal design, such as the recently published by Sarabia-Montaño et al. (2025), to investigate whether higher BF predicts better functional or psychological outcomes [45]. Second, despite we involved a large sample and enrolled from different MS centres, we have not fully captured the diversity of PwMS and caregivers in terms of age, socio-economic, cultural and disease-related factors. Finally, future studies should investigate BFS ability to detect change over time (responsiveness) as well as its predictive validity.

To conclude, the Italian version of BFS demonstrated strong psychometric properties and measurement invariance across PwMS and their caregivers, supporting its use as a multidimensional instrument for assessing BF in both populations. Its use in clinical practice and research could help health professionals track participants’ experience of positive changes under adverse circumstances, as assets in managing stress and promoting illness adjustment.
