# Correction to: Assessing validity and reliability of the Benefit Finding Scale in Italian people with multiple sclerosis and their caregivers

**DOI:** 10.1007/s10072-026-08880-1

**Published:** 2026-02-07

**Authors:** Rosalba Rosato, Andrea Giordano, Beatrice Biolzi, Clara Grazia Chisari, Monica Falautano, Monica Grobberio, Claudia Niccolai, Erika Pietrolongo, Maria Esmeralda Quartuccio, Rosa Gemma Viterbo, Antonella Delle Fave, Marta Bassi

**Affiliations:** 1 Department of Psychology, University of Turin, Turin, Italy; 2 Neurology, Public Health, Disability Unit, Fondazione IRCCS Istituto Neurologico C. Besta, Milan, Italy; 3 Multiple Sclerosis Center, Neurology Unit, Hospital of Vaio, Fidenza, Italy; 4 Dipartimento di Scienze Mediche e Chirurgiche e Tecnologie Avanzate, GF Ingrassia, University of Catania, Catania, Italy; 5 Psychological Service - Neurological and Neurological Rehabilitation Units, IRCCS San Raffaele, Milano, Italy; 6 Laboratory of Clinical Neuropsychology, Psychology Unit, ASST Lariana, Como, Italy; 7 IRCCS Don Gnocchi Foundation, Florence, Italy; 8 Department of Neurosciences, Imaging and Clinical Sciences, University G. d’Annunzio, Chieti, Italy; 9 Department of Neuroscience, San Camillo-Forlanini Hospital, Rome, Italy; 10 Department of Basic Medical Sciences, Neurosciences and Sense Organs, University of Bari, Bari, Italy; 11 Department of Pathophysiology and Transplantation, Università di Milano, Milan, Italy; 12 Department of Biomedical and Clinical Sciences, Università di Milano, Milan, Italy

**Correction to: Neurological Sciences**

10.1007/s10072-025-08776-6

In the original version of this article, the original corresponding author Dr. Rosalba Rosato should be reinstated as the first author, as her designation was inadvertently removed in the final version.

Additionally, the 1st and 2nd authors should be swapped to match the order presented in the manuscript and Dr. Andrea Giordano should be the corresponding author.

The original article has been corrected.

